# Identification of attention-deficit hyperactivity disorder based on the complexity and symmetricity of pupil diameter

**DOI:** 10.1038/s41598-021-88191-x

**Published:** 2021-04-19

**Authors:** Sou Nobukawa, Aya Shirama, Tetsuya Takahashi, Toshinobu Takeda, Haruhisa Ohta, Mitsuru Kikuchi, Akira Iwanami, Nobumasa Kato, Shigenobu Toda

**Affiliations:** 1 Department of Computer Science, Chiba Institute of Technology, 2-17-1 Tsudanuma, Narashino, Chiba 275-0016 Japan; 2 Department of Preventive Intervention for Psychiatric Disorders, National Institute of Mental Health, National Center of Neurology and Psychiatry, Tokyo, Japan; 3 Research Center for Child Mental Development, Kanazawa University, Ishikawa, Japan; 4 Department of Neuropsychiatry, University of Fukui, Fukui, Japan; 5 Uozu Shinkei Sanatorium, Uozu, Japan; 6 Faculty of Letters, Ryukoku University, Kyoto, Japan; 7 Medical Institute of Developmental Disabilities Research, Showa University, Tokyo, Japan; 8 Department of Psychiatry and Behavioral Science, Kanazawa University, Ishikawa, Japan; 9 Department of Psychiatry School of Medicine, Showa University, Tokyo, Japan; 10 Department of Psychiatry, Showa University East Hospital, Showa University, Tokyo, Japan

**Keywords:** Computational biology and bioinformatics, Neuroscience, Biomarkers

## Abstract

Adult attention-deficit/hyperactivity disorder (ADHD) frequently leads to psychological/social dysfunction if unaddressed. Identifying a reliable biomarker would assist the diagnosis of adult ADHD and ensure that adults with ADHD receive treatment. Pupil diameter can reflect inherent neural activity and deficits of attention or arousal characteristic of ADHD. Furthermore, distinct profiles of the complexity and symmetricity of neural activity are associated with some psychiatric disorders. We hypothesized that analysing the relationship between the size, complexity of temporal patterns, and asymmetricity of pupil diameters will help characterize the nervous systems of adults with ADHD and that an identification method combining these features would ease the diagnosis of adult ADHD. To validate this hypothesis, we evaluated the resting state hippus in adult participants with or without ADHD by examining the pupil diameter and its temporal complexity using sample entropy and the asymmetricity of the left and right pupils using transfer entropy. We found that large pupil diameters and low temporal complexity and symmetry were associated with ADHD. Moreover, the combination of these factors by the classifier enhanced the accuracy of ADHD identification. These findings may contribute to the development of tools to diagnose adult ADHD.

## Introduction

Attention-deficit/hyperactivity disorder (ADHD) is a neurodevelopmental disorder characterized by inattention, impulsivity, and hyperactivity^1,2^. As ADHD develops, the deficits of attentional function mostly remain, while impulsivity and hyperactivity become less apparent^3–6^. Therefore, it is more difficult to detect the symptoms of ADHD in adults than in children^7–9^. Moreover, adults with ADHD frequently experience psychological and social dysfunctions without appropriate treatment^10–12^. Thus, the advent of any biomarker to support objective and quantitative diagnosis of adult ADHD is desirable (reviewed in^13^)^3,4,14^.

Neuro-imaging methods, such as electroencephalogram (EEG) and functional magnetic resonance imaging (fMRI), have revealed abnormal neural activity associated with attentional functions in patients with ADHD (reviewed in^15–18^)^19,20^. This is caused by overactivity in the locus coeruleus (LC)^21–23^, which is a norepinephrine (NE) neural pathway that spans the entire brain and plays a critical role in arousal and attention (reviewed in^24^). In addition to overactivity of LC, the dysfunction of the right hemispheric LC, which has a crucial role in attention functions^25^, has been pointed^26^. However, it is difficult to measure LC activity directly using EEG because the region is located deep inside the brainstem. In contrast, fMRI cannot capture detailed LC activity over time because it has low temporal resolution.

Baseline LC activity is strongly reflected in pupil diameter^27–29^ (review in^30^) because the LC is the common source for both the sympathetic pathway to the pupil dilator muscle and the parasympathetic pathway to the pupil sphincter muscle^31,32^. Therefore, pupil diameter can indicate deficits in attention or arousal, as well as imbalances in the exploration–exploitation trade-off in several psychiatric disorders, including schizophrenia^33,34^ and autism spectrum disorder (ASD)^35–38^. In particular, since the deficits of attention and arousal functions are the core symptoms of ADHD, the behaviour of pupil diameter may directly reflect the symptoms/pathology of ADHD^39,40^. Furthermore, capturing the behavior of pupil diameter using the eye tracker is highly effective in clinical practice because it is cost- and time-effective, widely available, and non-invasive^41–43^.

Accumulating evidence on the neural activity detected by EEG/magnetoencephalography (MEG)/fMRI demonstrates that temporal complexity and asymmetricity reflect cognitive functions^44,45^, healthy aging^46,47^, development^48,49^, and some psychiatric disorders (reviewed in^50,51^), such as ASD^52^, ADHD^53^, and schizophrenia^54,55^. In addition to EEG/MEG/fMRI studies, those using neural activity estimated by pupil behaviour to detect abnormal LC activity have focused on the complex temporal patterns of pupil diameter^56–58^ and asymmetricity of pupil diameters among both eyes^26,59^. In particular, Piu et al., Artoni et al., and Nakamura et al. showed that complex temporal patterns reflect attentional functions, arousal level, and the symptoms or pathology of psychiatric disorders^56–58^. Poynter demonstrated that pupil asymmetricity reflects attentional load, as well as ADHD symptoms of inattention and hyperactivity^26^.

**Figure 1:**
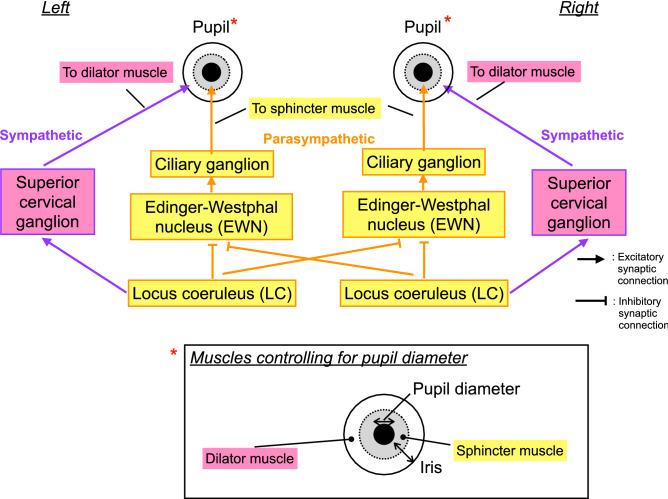
The neural pathways controlling pupil diameter. Detailed description for red * parts is shown in the box for the muscles controlling pupil diameter. This figure was created with the Keynote software version 10.3.9 (https://www.apple.com/keynote/).

To explain the mechanism underlying these pupil behaviours and their relationships with arousal and attention functions, mathematical models for the neural systems that control pupil diameters have been proposed^60–64^. In particular, as the first mathematical model, Usui and Stark constructed neural systems composed of the sympathetic pathway to the pupil dilator muscle and the parasympathetic pathway to the pupil sphincter muscle; these pathways were driven by common internal neural fluctuation^60^. Their study succeeded in modeling the autonomous temporal fluctuation of pupil diameter composed of frequency components from 0.04 to 2.0 Hz^65–67^, which is called hippus^60^. Recently Johansson and Balkenius developed a more complex pupil-controlling model comprising the amygdala, LC, cerebellum, and other regions; this model showed that various kinds of responses, including emotional and learning, in complex temporal patterns of pupil diameter appear as a consequence of cognitive tasks^64^. Furthermore, a recent study of pupil-controlling neural pathways by Liu et al. reported that the parasympathetic pathways from the LC project inhibitorily to both the ipsilateral and contralateral parts of the Edinger–Westphal nucleus (EWN) to control the pupil sphincter muscles, in addition to the ipsilateral parts as previously thought^68^ (see Fig. 1).

Regarding the recent trend of developing diagnosis tools for psychiatric disorders, several identification methods combining several feature values based on neural signals such as EEG/fMRI have been proposed and have exhibited high classification ability in psychiatric disorders^69–72^ (reviewed in^73^). However, the combination of pupil diameter features through machine learning to classify psychiatric disorders has not been developed, even though such an application might improve classification in cases where a single feature is inadequate. Moreover, the kinetics of pupil diameter may more directly reflect LC activity related to a deficit in arousal or attention, whereas the widely used EEG is based on neural activity measured from scalp electrodes. In this context, we hypothesized that analysing the relationship among size, temporal patterns, and asymmetricity of pupil diameters will improve our understanding of deficits in controlling-pupil-diameter neural systems in adult ADHD. Consequently, classification methods that combine these features may contribute to the advent of a biomarker for adult ADHD.

In the present study, to validate our hypothesis, we evaluated the hippus of the resting state in the adult subjects with or without ADHD. Specifically, we analysed the relationship between baseline pupil activity during the hippus and sample entropy (SampEn)^74^ as a measure of a temporal complexity. Moreover, we analysed transfer entropy (TranEn)^75^ as a measure of the asymmetricity of left and right pupil diameters. Based on these features, we constructed a classifier using logistic regression and evaluated the ability of classification between healthy adult subjects (typical development [TD]) and subjects with ADHD.

## Material and methods

### Participants

This study included 16 subjects diagnosed with adult ADHD and 20 age-, sex-, and intelligence-matched TD subjects^40^. The sample size for this study was determined based on the sample size used in previous studies regarding the relationship between pupil diameter and psychiatric disorders—including ADHD^33,76,77^. To assess their intelligence levels, all participants were evaluated with the Wechsler Adult Intelligence Scale-Third Edition, revised Japanese edition (WAIS-III), which comprises the intelligence quotient (IQ), verbal IQ (VIQ), and performance IQ (PIQ). To assess the subjects’ ADHD symptoms, they were subjected to the Japanese version of the adult ADHD Self-Report Scale (ASRS)^78^.

Subjects with ADHD were recruited from outpatient consultations at Seiwa Hospital, Tokyo, Japan. They were diagnosed based on the criteria of the Diagnostic and Statistical Manual of Mental Disorders, Fifth Edition^2^ through a semi-structured interview: the Assessment System for Individuals with ADHD^79^. The ADHD group consisted of 11 subjects under drug-naïve conditions and five subjects treated with methylphenidate (MHP; average dose, 45 mg/day) or atomoxetine (ATX; average dose, 80 mg/day). These five subjects stopped taking their ADHD medication on the experimental day. In this study, we defined 1-day medicine-free as being almost equal to drug-naïve, a paradigm which has been used in previous studies^39,40,80^, because the average half-lives of methylphenidate and atomoxetine are 3.5 and 5 h, respectively. The participants with ADHD included ten primarily inattentive (ADHD/I) subjects and six combined inattentive/hyperactive (ADHD/C) subjects. To remove the influence of ADHD medications, a drug-naïve ADHD group was created by excluding the drug-treated subjects from the ADHD group.

None of the TD subjects displayed clinically significant levels of ADHD symptomatology, as indexed by the ASRS. In both groups, we set the following exclusion criteria: a current major depressive or manic-depressive episode, a history of psychosis, Wechsler full-scale intelligence quotient $< 80$, a history of head injury with loss of consciousness, a sensory-motor handicap, or other neurological illnesses. All participants had normal or corrected-to-normal vision and normal hearing. The subjects’ detailed information is described in Table 1. The study was conducted at the Medical Institute of Developmental Disabilities Research, Showa University, Japan. After receiving a complete explanation of the study, all participants provided written informed consent. All methods were carried out in accordance with the Declaration of Helsinki, and the study protocol was approved by the Ethics Committee of Showa University.

**Table 1:** Physical characteristics in typical development (TD) and attention-deficit/hyperactivity disorder (ADHD) subjects.

	TD	ADHD	drug-naïve ADHD	*p*-values (TD vs. ADHD)	*p*-values (TD vs. drug-naïve ADHD)
Male/female	8/12	8/8	4/7	0.73	1.00
Age (year)	$37.0\;(7.90$, 22–51)	$32.0\;(8.29$,23–50)	$28.5\;(4.41$, 23–38)	0.077	${\mathbf {0.002}}$
FIQ score	$104.8\;(11.0$, 87–126)	$102.0\;(14.3$, 79–126)	$105.7\;(13.36$, 90–126)	0.523	0.837
VIQ score	$103.7\;(10.6$, 84–120)	$103.4\;(13.8$, 81–124)	$106.3\;(12.31$, 90–124)	0.949	0.530
PIQ score	$105.1\;(12.6$, 79–123)	$97.8\;(16.4$, 65–125)	$100.7\;(16.31$, 76–125)	0.142	0.840
ASRS					
Total score	$20.4\;(11.0$, 3–38)	$42.9\;(14.0$, 14–60)	$40.6\;(11.11$, 25–57)	${\mathbf {<0.001}}$	${\mathbf {<0.001}}$
ASRS					
IN score	$12.5\;(6.64$, 3–24)	$25.5\;(6.86$, 9–33)	$24.7\;(5.06$, 17–33)	${\mathbf {<0.001}}$	${\mathbf {<0.001}}$
ASRS					
Hyp/I score	$8.30\;(5.42$, 0–19)	$17.6\;(6.86$, 4–33)	$16.27\;(7.76$, 4–25)	${\mathbf {<0.001}}$	${\mathbf {0.002}}$

The group, which consists of ADHD subjects on medication and drug-naïve ADHD subjects, is represented by the ADHD group; the group which consists of only drug-naïve ADHD subjects, is represented by drug-naïve ADHD group. For group comparison sex ratio, the $\chi ^2$-test was used. For the other group comparisons, a two-tailed *t*-test was used. The *p*-values with $p < 0.05$ are represented by bold text. (FIQ, full-scale intellectual quotient; VIQ, verbal intellectual quotient; PIQ, performance intellectual quotient; ASRS, Japanese version of the adult ADHD self-report scale; ASSRS IN, ASRS inattention; ASRS Hyp/I, ASRS hyperactivity/impulsivity).

### Recording pupil diameters

To measure the pupil diameters, subjects sat in front of a monitor subtending $50.9^{\circ } \times 28.6^{\circ }$ of visual angle at 57 cm distance in a lit room. The subjects’ head position was fixed by a chin-rest. For 2 min, the subjects fixed their gaze at a steady black cross ($0.87\,{\text {cd/m}}^2$) subtending $0.5^{\circ }\times 0.5^{\circ }$ of visual angle to obtain the pupil diameter during hippus. The gaze objectives were generated using the Psychophysics Toolbox routines^81,82^ for MATLAB (Version 2013b; MathWorks Ltd, http://www.mathworks.com/) and presented on a 23-in. LCD monitor ($1920 \times 1080$ pixels at 60 Hz) driven by a computer running Windows 7. During these measurements, the subject’s eye position and pupil diameter were observed by a remote-type eye tracker (TX300; Tobii Technology, Stockholm, Sweden) with a sampling frequency of 300 Hz. While obtaining the pupil diameter, the TX300 can measure the distance between the eyes and eye tracker. On the day of the experiment, subjects ingested no caffeine, nicotine, or any medication that could influence eye movements or pupil diameter.

**Figure 2:**
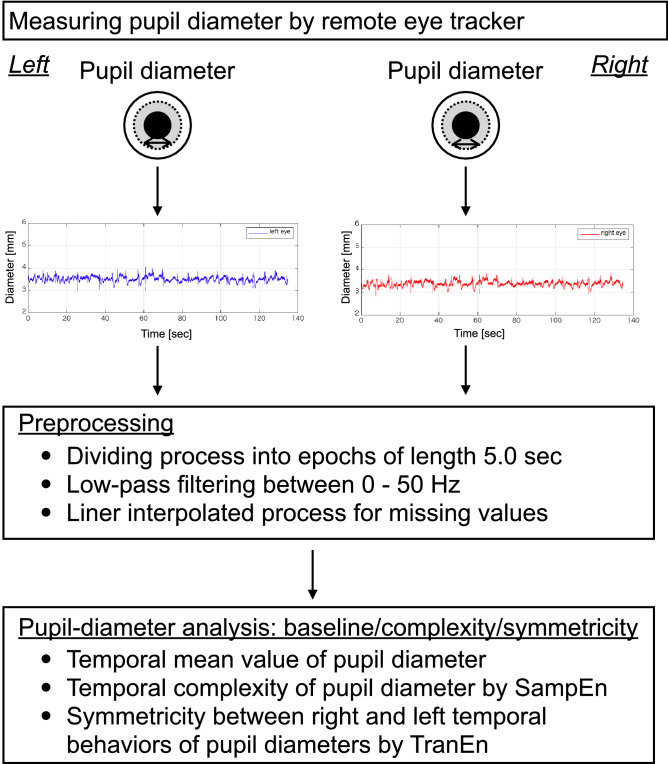
Conceptual figure regarding the measurement of pupil diameters, this preprocessing, and analysis for baseline, complexity, and symmetricity of pupil diameters. The figure was created with the Keynote software version 10.3.9 (https://www.apple.com/keynote/). Figures of time-series of pupil diameters were drawn by MATLAB R2019b (http://www.mathworks.com/).

To analyse pupil diameters, as preprocessing, the time-series of pupil diameters were divided into epochs with lengths of 5.0 s in preprocessing. The missing values in epoch were linearly interpolated. The epochs were low-pass filtered between 0 and 50 Hz. Conceptual figure regarding the measurement of pupil diameters, this preprocessing, and analysis for baseline, complexity, and symmetricity of pupil diameters (see “Analysis of baseline pupil diameters”, “Analysis of complexity of pupil diameters”, and “Analysis of symmetricity of pupil diameters”) are shown in Fig. 2.

### Analysis of baseline pupil diameters

To quantify the pupil diameters during baseline activity, we used the temporal mean value of the pupil diameters. These values were averaged between the right and left eyes and subsequently among epochs.

### Analysis of complexity of pupil diameters

We evaluated the temporal complexity of pupil diameter using sample entropy (SampEn). SampEn has been widely used to analyse the complexity of temporal patterns with high temporal resolution in the time-series of neural activity^55,72,83^. Against *N* stochastic variables $\{x_1,x_2,\ldots x_N\}$ normalized by z-score, the following *m*-dimensional vectors were constructed:

1 $$\begin{aligned} {\mathbf {x}}^m_i=\{x_i,x_{i+1},\ldots ,x_{i+m-1}\}. \end{aligned}$$

Against $i,j (i\ne j$, $i,j=1,2,\ldots )$ pairs of these vectors, the probability $C_m(r)$ is calculated by

2 $$\begin{aligned} C_m(r) = \sum _{i, j \in r, i \ne j} \frac{|{\mathbf {x}}^m_i - {\mathbf {x}}^m_j |}{(N-m+1)(N-m)}. \end{aligned}$$

here, $\sum _{i, j \in r, i \ne j} |{\mathbf {x}}^m_i - {\mathbf {x}}^m_j |$ means that the number of vector pairs is counted when the distance between two vectors ${\mathbf {x}}^m_i$ and ${\mathbf {x}}^m_j$ as the norm is less than tolerance *r*. SampEn *h*(*r*, *m*) was defined as

3 $$\begin{aligned} h(r,m)=-\log \frac{C_{m+1}(r)}{C_m(r)}, \end{aligned}$$

where we used $r=0.2,m=2$^84^. If the time-series becomes complex, *h*(*r*, *m*) exhibits a large value. The values of *h*(*r*, *m*) were averaged among epochs. In this study, PhysioToolkit (http://physionet.incor.usp.br/physiotools/sampen/), a toolbox in MATLAB$^{{\textregistered }}$^85^, was used for the calculation of SampEn.

### Analysis of symmetricity of pupil diameters

To analyse the symmetricity of the right and left pupil diameters, we ascertained causality between the right and left temporal behaviours of the pupil diameters using transfer entropy (TranEn). The TranEn $T_{X\rightarrow Y}$ from the time-series $\{x_1,x_2,\ldots ,x_t,\ldots \}$ to $\{y_1,y_2,\ldots ,y_t,\ldots \}$ is defined as follows^75^:

4 $$\begin{aligned} T_{X\rightarrow Y}=\sum _{y_{t+\tau },{{\mathbf{y}}_{\mathbf{t}}}^{d_y},{{\mathbf{x}}_{\mathbf{t}}}^{d_x}} p(y_{t+\tau }|{{\mathbf{y}}_{\mathbf{t}}}^{d_y},{{\mathbf{x}}_{\mathbf{t}}}^{d_x})\log \left( \frac{p(y_{t+\tau }|{{\mathbf{y}}_{\mathbf{t}}}^{d_y},{{\mathbf{x}}_{\mathbf{t}}}^{d_x})}{p(y_{t+\tau }|{{\mathbf{y}}_{\mathbf{t}}}^{d_y})}\right) , \end{aligned}$$

where *t* is a time-index, $t+\tau$ exhibits the prediction time, and $p(\cdot |\cdot )$ is the conditional probability. ${{\mathbf{y}}_{\mathbf{t}}}^{d_y}$ and ${{\mathbf{x}}_{\mathbf{t}}}^{d_x}$ denotes $d_x$- and $d_y$-dimensional delay vectors:

5 $$\begin{aligned} {{\mathbf{x}}_{\mathbf{t}}}^{d_x}= & {} \left( x_t,x_{t-\tau },\ldots ,x_{t-(d_x-1)\tau }\right) , \end{aligned}$$

6 $$\begin{aligned} {{\mathbf{y}}_{\mathbf{t}}}^{d_y}= & {} \left( y_t,y_{t-\tau },\ldots ,y_{t-(d_y-1)\tau }\right) . \end{aligned}$$

In a case with no causality from *X* to *Y*, $T_{X\rightarrow Y}=0$. With increasing causality from *X* to *Y*, $T_{X\rightarrow Y}$ increases in value. High TranEn corresponds to symmetricity of pupil diameters, whereas low TranEn corresponds to asymmetricity. The TranEn values were averaged between the right-to-left and left-to-right pupil diameters. The values were then averaged among epochs. The frequency component of hippus distributes from 0.04 to 2.0 Hz^65–67^. Therefore, the delay needed to be set sufficiently faster than 2.0 Hz. Additionally, the conditional probability in Eq. (4) had to be estimated. For this estimation, a sufficient sample size of delay vectors given by Eqs. (5) and (6) was needed. Consequently, TranEn was measured from the right to left and from the left to right pupil diameters using $\tau =10$ (corresponding period, 0.033 s). To evaluate the behaviours of pupil diameter including slow frequency components, dimensions for delay vectors was set to as large as possible to allow the estimation of conditional probability in Eq. (4); consequently, we used $d_x=d_y=5$. In this study, HERMES a toolbox in MATLAB$^{{\textregistered }}$, was utilized to calculate TranEn^86^.

### Surrogate data analysis

To investigate whether a non-linear dynamic process was involved in the temporal behaviours of pupil diameters in both TD and ADHD, we used iterative, amplitude-adjusted Fourier transformed (IAAFT) surrogate data analysis^87^ with an iteration number of 50. Ten IAAFT surrogate datasets were generated using different random seeds per original pupil diameter. These SampEn and TranEn values were averaged and compared with the value from the original pupil diameters.

### Statistical analysis

To compare the SampEn and TranEn values from the original pupil diameter time-series with those from the IAFFT surrogate time-series, we used a paired *t*-test. To compare the size, SampEn, and TranEn of pupil diameter between the TD and ADHD groups, we used analysis of covariance (ANCOVA) with age as a covariate. To assess the significant main effect of group, post hoc *t*-tests were used. In *t*-tests, a two-tailed $\alpha$ level of 0.05 was defined as statistically significant. Using Pearson’s correlation coefficient, we evaluated how the severity and ADHD symptoms, measured using ASRS scores, were related to the size, SampEn, and TranEn of the pupil diameter. To this end, we used the total ASRS score and the ASRS subscores; that is, ASRS of hyperactivity/impulsivity (ASRS Hyp/I) score and ASRS of inattentive (ASRS IN) score.

To identify ADHD, a logistic regression based on the SampEn, TranEn, and size of pupil diameter was used. Pearson’s correlation *R* among SampEn, TranEn, and size was used for multicollinearity in the logistic regression. For the criteria of multicollinearity, $|R|>0.8$ was set. To evaluate the classification ability of TD and ADHD groups by logistic regression and measure the balance between sensitivity and specificity, the receiver operating characteristics (ROC) curve was used^88^. To obtain ROC curves, the probability for ADHD estimated by the logistic regression was utilized. By changing the threshold of this probability to judge ADHD from 0 to 1.0, true-positive and false-positive rates were calculated at each threshold. The performance based on the ROC curve was quantified by the area under the ROC curve (AUC) to determine the overall identification accuracy. AUCs of 1.0 and 0.5 correspond to the case for perfect discriminating ability and the case for random prediction, respectively. For the logistic regression, we used the function for a generalized linear regression model in the Statistics and Machine Learning Toolbox of MATLAB$^{{\textregistered }}$.

## Results

**Figure 3:**
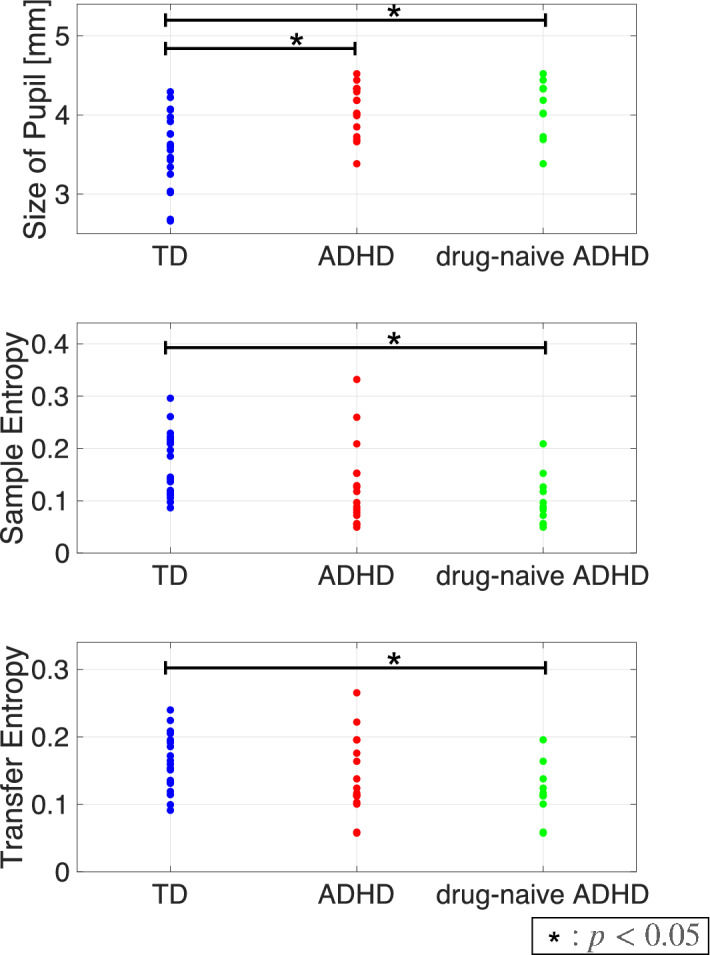
Pupil diameter size, sample entropy (SampEn), and transfer entropy (TranEn) in TD group, ADHD group which comprised subjects on medication and drug-naïve subjects, and drug-naïve ADHD groups. The size of the pupil diameters and SampEn were averaged between the right and left pupils, whereas the values of TranEn were averaged between the cases from right to left and left to right. Each dot corresponds to size/SampEn/TranEn for each subjects. The comparison between the TD and ADHD groups indicated significant larger pupil diameter in the ADHD group. In a comparison between the TD and drug-naïve ADHD groups, there was significantly larger pupil diameter and smaller SampEn and TranEn in the drug-naïve ADHD group. These statistical values are shown in Tables 3 and 4. This figure was drawn by MATLAB R2019b (http://www.mathworks.com/) and the Keynote software version 10.3.9 (https://www.apple.com/keynote/).

First, an IAAFT surrogate data analysis against SampEn and TranEn was conducted (Table 2). The IAAFT procedure produced significant enhancements in complexity and symmetricity. That is, the SampEn and TranEn of pupil diameter showed that the behaviour of pupil diameters reflected a deterministic process in all groups. Second, the size, complexity, and symmetricity of pupil diameters were evaluated in the TD group, the ADHD group consisting of subjects on medication and drug-naïve subjects, and the drug-naïve ADHD group. Table 3 summarizes the results of the ANCOVA with age as a covariate against the size, SampEn, and TranEn of pupil diameter. There was a significant main effect of group in size in the comparison between TD and ADHD, as well as a significant main effect of size, SampEn, and TranEn in the comparison between TD and drug-naïve ADHD. Table 4 summarizes the post hoc *t*-test of ANCOVA. The comparison between the TD and ADHD groups indicated that pupil diameter was significantly larger in the ADHD group. The comparison between the TD and drug-naïve ADHD groups showed that pupil diameter was significantly larger in the drug-naïve ADHD group, whereas complexity and symmetricity were significantly smaller. The values of size, SampEn, and TranEn in each subject are represented in Fig. 3.

**Table 2:** Surrogate data analysis of pupil diameter temporal complexity measured using sample entropy (SampEn) and pupil diameter symmetricity measured using transfer entropy (TranEn) in the TD and ADHD groups.

	Original (mean, [SD])	Surrogate data (mean, [SD])	*t*- (*p*-) value
**TD**			
SampEn	$0.170\;(0.060)$	$0.254\;(0.056)$	${\mathbf {6.38\;(<0.001)}}$
TranEn	$0.160\;(0.041)$	$0.208\;(0.038)$	${\mathbf {6.10\;(<0.001)}}$
**ADHD**			
SampEn	$0.128\;(0.079)$	$0.207\;(0.090)$	${\mathbf {5.42\;(<0.001)}}$
TranEn	$0.141\;(0.057)$	$0.202\;(0.063)$	${\mathbf {5.56\;(<0.001)}}$
$${\mathbf{{Drug\text{-} \mathbf{na\"ive}} \; ADHD}}$$			
SampEn	$0.100\;(0.048)$	$0.194\;(0.074)$	${\mathbf {5.57\;(0.002)}}$
TranEn	$0.117\;(0.040)$	$0.186\;(0.037)$	${\mathbf {6.36\;(<0.001)}}$

SampEn and TranEn of the original time-series and of the corresponding iterative, amplitude-adjusted, Fourier transformed (IAAFT) surrogate time-series. Mean (standard deviation, SD) values within groups are represented. The surrogate process showed significant enhancements in SampEn and TranEn. The *t*- and *p*- values with $p < 0.05$ are represented by bold text.

**Table 3:** Analysis of covariance (ANCOVA) of the pupil diameter temporal mean values, described as the pupil diameter size, temporal complexity of pupil diameters measured by SampEn, and symmetricity of pupil diameters measured by TranEn, with group (TD group vs. ADHD group, TD vs. drug-naïve ADHD) as the between-subject factor and age as the covariate.

	TD vs. ADHD	TD vs. drug-naïve ADHD
Group effect for size	${\mathbf {F=9.04,p<0.005,\,}}{\mathbf {\eta }}^{\mathbf {2}}{\mathbf {=0.215}}$	${\mathbf {F=4.19,p<0.05,\,}}{\mathbf {\eta }}^{\mathbf {2}}{\mathbf {=0.13}}$
Group effect for SampEn	$F=1.55,p=0.22,\eta ^2=0.05$	${\mathbf {F=7.23,p<0.012,\,}}{\mathbf {\eta }}^{\mathbf {2}}{\mathbf {=0.21}}$
Group effect for TranEn	$F=0.22,p=0.65,\eta ^2=0.007$	${\mathbf {F=4.19,p<0.05,\,}}{\mathbf {\eta }}^{\mathbf {2}}{\mathbf {=0.13}}$

The significant group difference ($p<0.05$) is represented by bold text.

**Table 4:** Post-hoc *t*-test for group comparisons of pupil diameter, SampEn, and TranEn, as well as their mean and SD values in each group.

	TD (mean, [SD])	ADHD (mean, [SD])	drug-naïve ADHD (mean, [SD])	*t*- (*p*-) values (ADHD vs. TD)	*t*- (*p*-) values (drug-naïve ADHD vs. TD)
Size (mm)	$3.56\;(0.469)$	$4.06\;(0.324)$	$4.08\;(0.360)$	${\mathbf {3.56\;(0.001)}}$	${\mathbf {3.19\;(0.003)}}$
SampEn	$0.170\;(0.060)$	$0.128\;(0.079)$	$0.100\;(0.048)$	$-1.80\;(0.078)$	${\mathbf {-3.30\;(0.002)}}$
TranEn	$0.160\;(0.041)$	$0.141\;(0.057)$	$0.117\;(0.040)$	$-1.17\;(0.249)$	${\mathbf {-2.76\;(0.009)}}$

Significant group difference ($p < 0.05$), is indicated by bold text. Positive *t*-values correspond to larger values among the subjects with ADHD than among the TDs.

**Figure 4:**
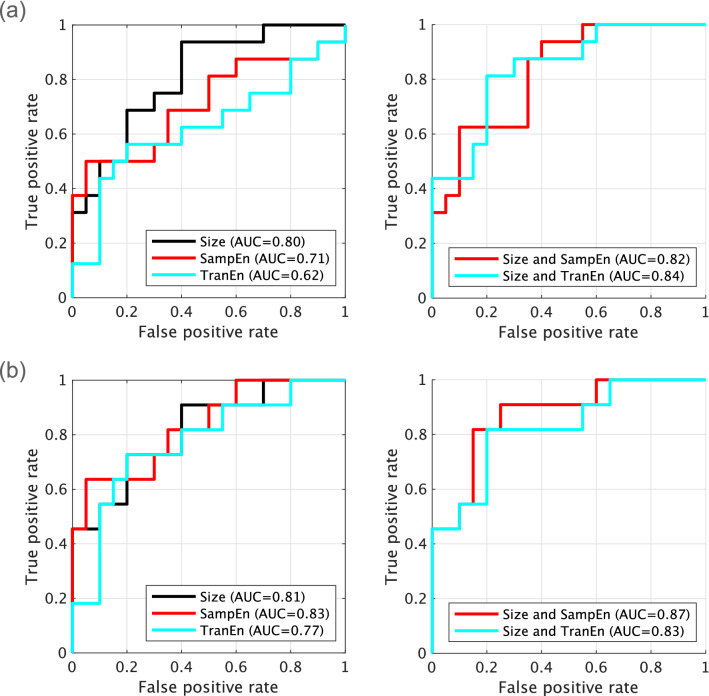
Receiver operating characteristic (ROC) curve for classifiers of TD/ADHD by logistic regression based on size, SampEn, and TranEn of pupil diameters. The cases of classifier separately using size, SampEn, and TranEn (left part) and the cases of classifier combining size/SampEn and size/TranEn (right part). The area under the ROC curve (AUC) was enhanced by the combination of size, SampEn, and TranEn in comparison with cases that used them separately. (**a**) Classifiers in the TD/ADHD groups. (**b**) Classifiers in the TD and drug-naïve ADHD groups. This figure was drawn by MATLAB R2019b (http://www.mathworks.com/).

Furthermore, classifiers in the TD and ADHD groups were evaluated by size, complexity, and symmetricity of pupil diameters. Figure 4a shows the result for ROC analysis of TD/ADHD by logistic regression based on the size, SampEn, and TranEn values of pupil diameter. In the separate analyses using size, SampEn, and TranEn, the AUCs were 0.80, 0.71, and 0.62 (see left part of Fig. 4a). Furthermore, to evaluate the combination of size, SampEn, and TranEn, the multicollinearity among them was checked by Pearson’s correlation *R* as shown in Table 5. The results showed that multicollinearity arises between SampEn and TranEn of pupil diameters. Therefore, the abovementioned combination was not used in the logistic regression. The combinations of size and SamEn and size and TranEn enhanced the AUC to 0.82 and 0.84, respectively (see right part of Fig. 4a). Figure 4b shows ROC curves in the case used classifier of TD/drug-naïve ADHD. In the cases separately using size, SampEn, and TranEn, the AUCs were 0.81, 0.83, and 0.77 (see left part of Fig. 4b). The multicollinearity was checked by Pearson’s correlation *R* as shown in Table 6. The result showed that multicollinearity arises in the combination of the SampEn and TranEn of pupil diameters. The combinations of size and SamEn and size and TranEn enhanced the AUC to 0.87 and 0.83, respectively, as well as the case of TD/ADHD (see right part of Fig. 4b).

**Table 5:** Pearson’s correlation coefficients *R* among the size, SampEn, and TranEn values of pupil diameters in TD and ADHD subjects.

	Size	SampEn	TranEn
Size	–	$R=-0.294\;(p=0.041)$	$R=-0.332\;(p=0.024)$
SampEn	–	–	${\mathbf {R=0.889\;(p<0.001)}}$

The *R*- and *p*-values with the criteria of multicollinearity $|R|>0.8$ are represented by bold text. The multicollinearity beween SampEn and TranEn emerges.

**Table 6:** Pearson’s correlation coefficients *R* among the size, SampEn, and TranEn values of pupil diameters in TD and drug-naïve ADHD subjects.

	Size	SampEn	TranEn
Size	–	$R=-0.376\;(p=0.019)$	$R=-0.425\;(p=0.009)$
SampEn	–	–	${\mathbf {R=0.882\;(p<0.001)}}$

The *R*- and *p*-values with the criteria of multicollinearity $|R|>0.8$ are represented by bold text. The multicollinearity beween SampEn and TranEn emerges.

**Figure 5:**
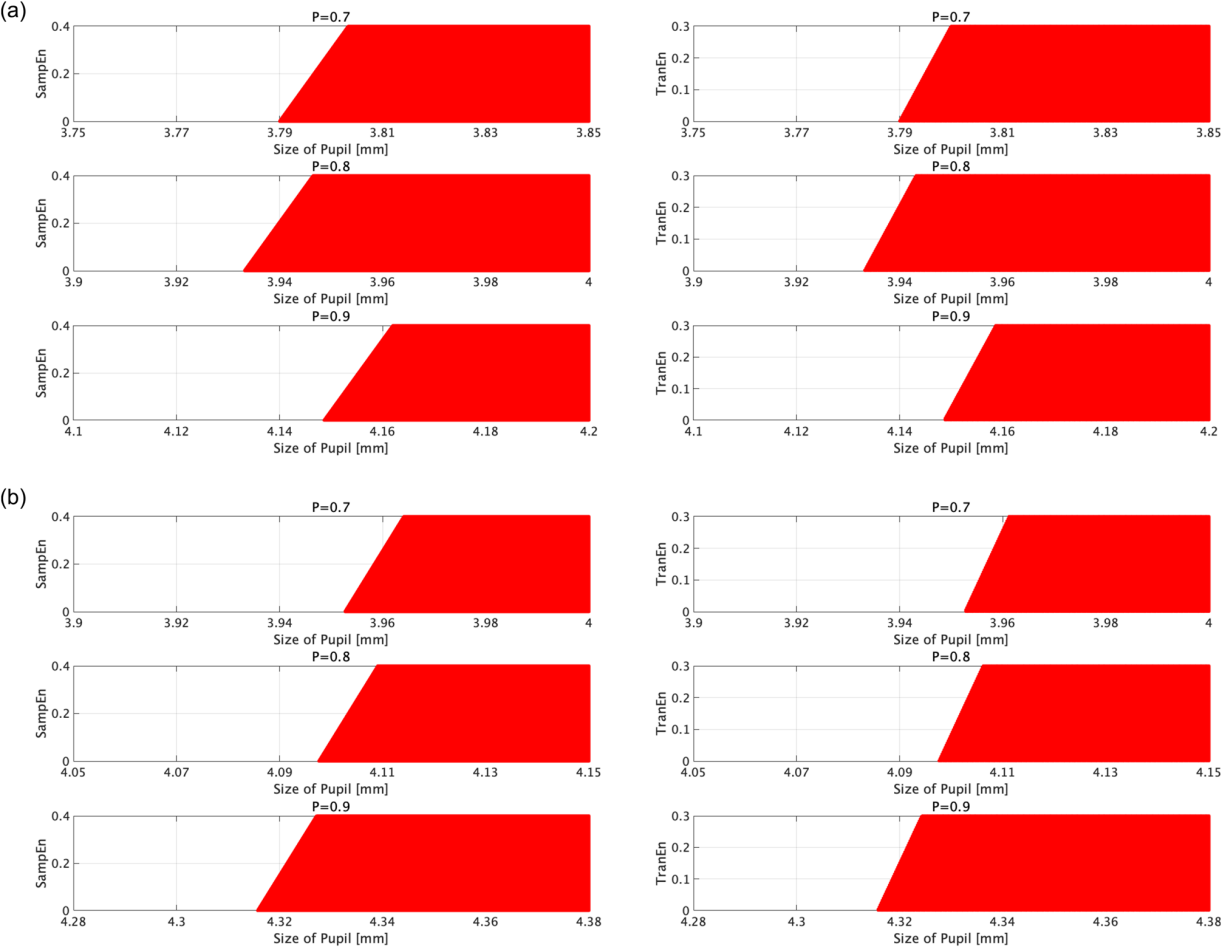
Decision regions for ADHD with probability *P* indicated by red dots on the size-SampEn plane in the classifier using size/SampEn and size-TranEn plane in the classifier using size/TranEn. The classifiers used the logistic regression learned by the combinations for size/SampEn, and the combination of size/TranEn of pupil diameters, which correspond to Fig. 4. (**a**) Classifier in the TD/ADHD groups. (**b**) Classifier in the TD/drug-naïve ADHD groups. This figure was drawn by MATLAB R2019b (http://www.mathworks.com/).

In the classifiers learned by the combinations for size/SampEn and for size/TranEn of the pupil diameters, we investigated the decision region for ADHD with probability *P* on the size-SampEn plane and size-TranEn plane in cases for classifier of TD/ADHD groups and TD/drug-naïve ADHD groups (see Fig. 5). The results showed that the decision region for ADHD depends on the size, SampEn, and TranEn. Hence, the classification abilities in the cases with combinations for size/SampEn and for size/TranEn (shown in Fig.4) are higher than those in the cases which used a single feature value among them.

**Figure 6:**
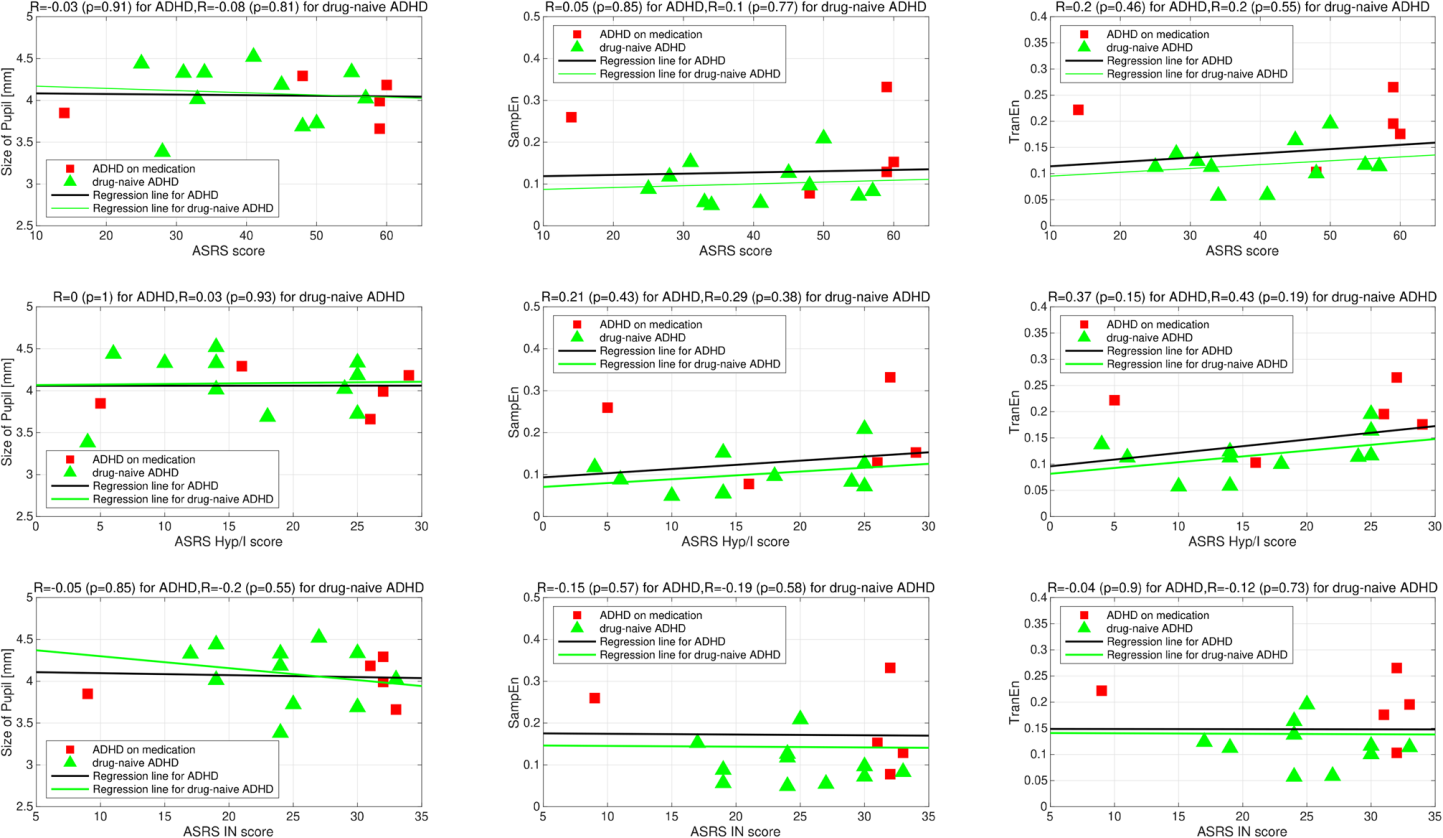
Scatter plots between size/SampEn/TranEn and score in the adult ADHD Self-Report Scale (ASRS; top). Scatter plots with ASRS subscores, that is, ASRS of hyperactivity/impulsivity (ASRS Hyp/I) score (middle) and ASRS of inattentive (ASRS IN) score (bottom). Pearson’s correlation coefficient *R* between size/SampEn/TranEn and ASRS score and the corresponding *p* value are represented in the upper part of each figure. *R* and regression lines are calculated in the case for ADHD group composed of drug-naïve subjects and subjects on medication and case for drug-naïve ADHD group. Size/SampEn/TranEn are not correlated with the severity and symptoms of ADHD in both groups. This figure was drawn by MATLAB R2019b (http://www.mathworks.com/).

Additionally, to investigate how size, SampEn, and TranEn were related to the severity and symptoms of ADHD, Fig. 6 shows scatter plots between size, SampEn, TranEn, and ASRS score, as well as the scatter plots for ASRS subscore, that is, ASRS of hyperactivity/impulsivity (ASRS Hyp/I) score and ASRS of inattentive (ASRS IN) score. The correlation coefficients (*R*) were low between the severity of ADHD (ASRS score) and size/SampEn/TranEn ($R\lesssim 0.2$) and between the symptoms of ADHD and size/SampEn/TranEn ($R\lesssim 0.43$ in ASRS Hyp/I score and $R\approx -0.15$ in ASRS IN score).

## Discussion

We evaluated the complexity and symmetricity of pupil diameter during hippus in the TD and ADHD groups. Surrogate data analysis revealed that the complexity and symmetricity of pupil diameter temporal behaviours reflected non-linear, deterministic processes in both groups. Pupil diameters are controlled by parasympathetic and sympathetic pathways; this implies that complexity and symmetricity are physiologically inherent to neural dynamics. Then, the size, complexity, and symmetricity of pupil diameters were compared between the TD and ADHD groups. In the ADHD group, the diameters were larger, and lower in complexity and symmetricity. Additionally, we constructed a classifier of TD/ADHD based on size, complexity, and symmetricity of pupil diameters. The results of the ROC evaluations indicated that the combinations of size/complexity and size/symmetricity of pupil diameters are associated with higher accuracy than their separate use. Finally, we investigated how size/complexity/symmetricity are related to the severity and symptoms of ADHD. The results showed that these factors are not correlated with the severity and symptoms of ADHD.

Regarding the size, temporal complexity, and symmetricity of pupil diameters in TDs and subjects with ADHD, subjects with ADHD had significantly larger pupil size than TDs, as shown in Table 4, perhaps because the LC is overactivated in ADHD^89,90^. Overactivation of the LC enhances the input of the dilator muscle and reduces that of the sphincter muscle^27–29^ (review in^30^) (see Fig. 1). Moreover, overactivity of the LC leads to reductions in pupil diameter complexity and symmetricity because parasympathetic pathways from the LC inhibit the activity of EWN, which controls the pupil sphincter muscles. Therefore, when the LC is overactivated, the pupil diameter is determined almost exclusively by the dilator muscle. Consequently, the temporal complexity is lower than when it is driven by both the parasympathetic and sympathetic pathways. Regarding symmetricity, LC overactivation inhibits the contralateral parts of the EWN, so the common inputs to the EWN between the right and left pupil are lost. Consequently, the symmetricity of the pupil diameters is reduced. In addition to LC overactivation, it was reported that the right hemispheric LC has a crucial role in attention functions^25^. Poynter demonstrated the possibility that subjects with high inattention and impulsivity/hyperactivity exhibited smaller right pupil diameter than the left one, i.e., reduction of symmetricity of the pupil diameters, due to the dysfunction of right hemispheric LC^26^. Thus, our results of reduction of symmetricity in ADHD are consistent with these findings. Moreover, in the present study, we observed significant reductions in complexity and symmetricity in ADHD when the TD and drug-naïve ADHD groups were compared. However, no such reductions were observed when the TD group and ADHD group including subjects on medication were compared (see Tables 3 and 4). A significant difference in pupil diameter was observed in both group comparisons. This result implies that complexity and symmetricity more strongly reflect the state of ADHD.

In determining ADHD, the advantage of evaluating complexity and symmetricity of pupil diameters by SampEn and TranEn, as used in this study, requires additional discussion. The conventional evaluation method for complexity and symmetricity of the behaviour of pupil diameters utilized temporal standard deviation, Shannon entropy, and size of difference between the right and left pupil diameters^26,56,59,60^. Piu *et al.* showed that, in addition to complexity estimated by Shannon entropy, the combination with determinism is effective as a diagnostic tool for identifying the states of neural systems^56^. Moreover, the importance of evaluation of determinism is supported by the fact that the behaviour of pupil diameters is produced from multiple non-linear neural pathways^60,68^. However, the temporal standard deviation, Shannon entropy, and the size of difference of pupil diameters cannot reflect determinism^88,91^. In contrast, SampEn and TranEn utilized in this study can precisely detect the determinism of pupil diameters in addition to the evaluations of complexity and symmetricity (see Table 2). Moreover, it was confirmed that the combinations for size/SampEn and for size/TranEn by machine learning enhance the accuracy of ADHD identification, in comparison with the cases using them separately (see Fig.4). Therefore, it can be assumed that the evaluation by SampEn and TranEn and machine learning with the combinations for size/SampEn and for size/TranEn might contribute to an implementation of a diagnostic tool for psychiatric disorders including ADHD.

The limitations of this study must be considered. In this study, as a preliminary study, the characteristics of size, temporal complexity, and symmetricity of pupil diameters in ADHD subjects were revealed in a relatively small sample size. It is necessary for our results to be validated whether these revealed characteristics emerge under different conditions with larger sample sizes, including a large variety of clinical backgrounds among subjects with ADHD. Moreover, the comparisons between the TD and ADHD groups showed different tendencies than the comparisons between the TD and drug-naïve ADHD groups, perhaps because the severity of ADHD differs between subjects on medication and those who are drug-naïve. However, an assessment of the severity before treatment could not be obtained in our study. Regarding the influence of medications, in this study, we defined 1-day medicine-free as being almost equal to drug-naïve. However, it remains possible that these medicines have already yielded some long-term pharmacological effects that endure for more than a few days due to their regular administration. Thus, 1-day off medicine may not be sufficient for an individual to be regarded as being genuinely medicine-free. This influence needs to be evaluated in future studies with a larger sample size consisting of both drug-naïve and drug-treated ADHD subjects. Evaluating the dependencies of pupil behaviours on the specific classification types of ADHD, i.e., ADHD/I, ADHD/hyperactive-impulsive, and ADHD/C types, is important; however, the sample size of this study was too small to allow for such analyses. Additionally, although our study revealed the complexity and symmetricity of autonomous pupil diameter behaviours in both TDs and subjects with ADHD, their variations under cognitive tasks and attention loads remain unclear. However, recent studies indicate that pupil response for cognitive task and light stimulus may reflect the pathology of psychiatric disorders, the performance of attention function, and estimation for sleep quality^33,38–40,92^. Applying our proposed method to these responses is important in determining its possible clinical application. Finally, in addition to pupil diameter in ADHD, researchers must evaluate similar profiles in other psychiatric disorders involving attention and arousal deficits or imbalances in the exploration–exploitation trade-off, such as schizophrenia and ASD. We intend to deal with these points in future studies.

## Conclusions

By analysing pupil diameters, we revealed that ADHD is associated with large pupil diameter and low complexity and symmetricity of dynamic pupil diameter behaviours. Moreover, the combination of these factors by machine learning enhances the accuracy of ADHD identification. Applying our proposed evaluation method and our findings may facilitate the development of tools to aid in ADHD diagnosis based on pupil diameter. Since they can indicate deficits in brain function and psychiatric disorders, our methods may be used for other pathologies.

## Data Availability

The datasets generated for this study will not be made publicly available because the informed patient consent did not include a declaration regarding public availability of clinical data. Requests to access the datasets should be directed to the corresponding author. Our developed source codes for analysis of size/complexity/symmetricity of pupil diameters and analysis for ROC/logistic regression can be found at the following address: https://github.com/SouNobukawa/pupil_TranEn_SampEn_size.
